# Tandem Repeats in Proteins: Prediction Algorithms and Biological Role

**DOI:** 10.3389/fbioe.2015.00143

**Published:** 2015-09-24

**Authors:** Marco Pellegrini

**Affiliations:** ^1^Laboratory for Integrative Systems Medicine (LISM), Istituto di Informatica e Telematica, and Istituto di Fisiologia Clinica, Consiglio Nazionale delle Ricerche, Pisa, Italy

**Keywords:** proteins, tandem repeats, biological significance, protein TR detection algorithms, protein TR properties

## Abstract

Tandem repetitions in protein sequence and structure is a fascinating subject of research which has been a focus of study since the late 1990s. In this survey, we give an overview on the multi-faceted aspects of research on protein tandem repeats (PTR for short), including prediction algorithms, databases, early classification efforts, mechanisms of PTR formation and evolution, and synthetic PTR design. We also touch on the rather open issue of the relationship between PTR and flexibility (or disorder) in proteins. Detection of PTR either from protein sequence or structure data is challenging due to inherent high (biological) signal-to-noise ratio that is a key feature of this problem. As early *in silico* analytic tools have been key enablers for starting this field of study, we expect that current and future algorithmic and statistical breakthroughs will have a high impact on the investigations of the biological role of PTR.

## Introduction

A seminal paper (Andrade et al., 2001) reports the observation that repetitive subsequences that appear in tandem repetitions (TR) within the protein primary sequence often form integrated assemblies when these residues are mapped to their corresponding three-dimensional folded conformation. These TR confer multiple binding opportunities and may play a structural role by giving rigidity to a protein, and by exposing functional domains. Moreover, Andrade et al. (2001) remark that *tandem repeated structures* should not be assimilated to the traditional notions of *domains* and *motifs* that may appear singly or in multiple interspersed copies in each protein (while they can be repeated across families of protein), since they constitute a rather distinct class. They also remark that repeats in protein sequences are usually hard to detect because on average the repeating unit is relatively short, and moreover there can be considerable sequence divergence among units of the same TR. We will refer throughout this article to these repetitive sub-sequences as *Protein Tandem Repeats* (PTR or Protein-TR, for short).

A study by Marcotte et al. (1998) indicates that internal subsequence repetitions in protein primary structure are quite widespread. They have been detected in about 14% of all the then known proteins, with eukaryotic proteins being three times more as likely to have internal repeats than prokaryotic ones. More recent measurements in (Pellegrini et al., 2012) give a count of about 25% of the proteins in the Uniprot database (Apweiler et al., 2004) holding a PTR of length at least 20 aa.

A recent survey of some algorithmic aspects of PTR detection in protein sequences is in Luo and Nijveen (2014). In this survey, we will touch lightly on the multi-faceted aspects of PTR research, including prediction algorithms, databases, early classification efforts, mechanisms of PTR formation and evolution, and synthetic PTR design. We also touch on the rather open issue of the relationship between PTR and flexibility (or disorder) in proteins.

## Protein-TR Detection Algorithms Based on Sequence

Structural and functional properties of Protein-TR are often preserved also in presence of high divergence among the subsequences corresponding to the PTR units, both at the level of DNA coding sequence and at the level of AA sequence. This property makes automatic PTR detection a challenging task, and a variety of approaches have been implemented since the late 1990s. More recently, a tendency to integrating basic sequence data with evolutionary or biochemical annotations has emerged. Table 1 reports the list of sequence-based algorithms.

**Table 1:** **Synthetic table of resources for PTR studies: sequence-based algorithms**.

Name	Type	Year	Reference	Notes
INTREP	Alg	1999	Pellegrini et al. (1999)	http://nihserver.mbi.ucla.edu/Repeats/
prospero	Alg	1999	Mott (1999)	http://www.well.ox.ac.uk/rmott/ARIADNE/prospero.shtml
REP	Alg	2000	Andrade et al. (2000)	http://www.bork.embl.de/~andrade/papers/rep/search.html
RADAR	Alg	2000	Heger and Holm (2000)	https://github.com/AndreasHeger/radar/
REPRO	Alg	2000	George and Heringa (2000)	http://www.ibi.vu.nl/programs/reprowww/
TRUST	Alg	2004	Szklarczyk and Heringa (2004)	http://www.ibi.vu.nl/programs/trustwww/
REPPER	Alg	2005	Gruber et al. (2005)	http://toolkit.tuebingen.mpg.de/repper/
HHrep	Alg	2006	Soding et al. (2006)	http://toolkit.tuebingen.mpg.de/hhrep
TRED	Alg	2006	Sokol et al. (2007)	Available upon request
XSTREAM	Alg	2007	Newman and Cooper (2007)	http://jimcooperlab.mcdb.ucsb.edu/xstream/
HHRepID	Alg	2008	Biegert and Soding (2008)	http://toolkit.tuebingen.mpg.de/hhrepid/
ARD2	Alg	2009	Palidwor et al. (2009)	http://cbdm.mdc-berlin.de/~ard2/
T-REKS	Alg	2009	Jorda and Kajava (2009)	http://bioinfo.montp.cnrs.fr/
REPETITA	Alg	2009	Marsella et al. (2009)	http://protein.bio.unipd.it/repetita/
PTRStalker	Alg	2012	Pellegrini et al. (2012)	http://bioalgo.iit.cnr.it/
TRDistiller	Alg	2014	Richard and Kajava (2014)	Available upon request

Interestingly, early algorithms by Marcotte et al. (1998), Pellegrini et al. (1999), and Andrade et al. (2000) were instrumental to the first PTR classification efforts, while more recent tools have been aimed at providing web-server-based utilities, or at populating databases.

REP in Andrade et al. (2000) is one of the first PTR detection algorithms which uses a homology-based method to identify statistically significant protein repeats.

Other early methods developed for finding TRs in proteins are based on detecting sub-optimal alignments in the self-alignment matrix generated by the Smith-Waterman algorithm (or similar methods). Some methods developed along this line are Internal Repeat Finder (Marcotte et al., 1998; Pellegrini et al., 1999), prospero (Mott, 1999), RADAR (Heger and Holm, 2000), REPRO (Heringa and Argos, 1993; George and Heringa, 2000), and TRUST (Szklarczyk and Heringa, 2004). These methods often detect both tandem and interspersed repeats.

XSTREAM (Newman and Cooper, 2007) uses a seed expansion approach, while Jorda and Kajava (2009) proposed T-REKS, which uses a clustering approach based on k-means.

The systems HHrep (Soding et al., 2006) and HHRepID (Biegert and Soding, 2008) are instead based on building and matching Hidden Markov Models for the repeating substrings to be sought (not necessarily tandem).

Some approaches based on neural networks aim at detecting particular repetitive structures. For example, Palidwor et al. (2009) developed a classification technique for detecting alpha-rods repeats, a specific important repetitive structure [see also Rubinson and Eichman (2012)].

For the class of protein solenoid repeats, REPETITA, by Marsella et al. (2009), uses several AA biochemical properties (including polarity, secondary structure, molecular volume, electric charge, and codon diversity) and a discrete fourier transform approach to detect self-similarities.

Pellegrini et al. (2012) propose the notion of *fuzzy TR* (FTR) for proteins, which is based on using a normalized BLOSUM-weighted edit distance between AA sub-strings and in assuming that in a FTR, even if the constitutive unit elements may be pairwise at high divergence, there exists an “origin” string, not necessarily still part of the protein in exam, that is at a relatively small divergence from any of its unit elements. Here, the notion of high/low divergence is relative to the divergence between random AA strings under the chosen weighted edit distance. An exhaustive search of FTRs in long proteins is computationally demanding, since the bare definition leads to an NP-hard problem. Thus, an efficient heuristic is used in *PTRStalker* to guess the candidate “origin” strings.

Gruber et al. (2005) propose REPPER a meta searching approach that combines the output of different algorithms. A web-based meta-search server that allows to run and compare easily several tools on the same input is also described in Schaper et al. (2015).

Shapper et al. (Schaper et al., 2012; Anisimova et al., 2015) propose a statistical method based on phylogenetic fingerprints and ML-estimation that, in conjunction with one or more standard predictors, is able to filter out predicted TR that are more likely to be false-positive.

As screening large portions of protein sequence DB looking for TR patterns is time consuming, Richard and Kajava (2014) propose a pre-screening tool (TRDistiller) whose purpose is to quickly filter out proteins that almost surely do not contain a TR, while retaining for further analysis the proteins carrying a TR with high probability.

As the list of possible tools to choose from becomes longer, there is an emerging need for guidance on which tool is most suitable for a given task. Unfortunately, at the best of my knowledge, no such comprehensive comparative study has been attempted yet. More limited comparative tests can be found in Pellegrini et al. (2012) where five methods (RADAR, TRUST, T-REKS, XSTREAM, and PTRStalker) are compared in their ability to detect very long PTRs (≥ 4000 AA), with XSTREAM and PTRStalker emerging as the best choice for this task. A second test is aimed at detecting dimeric proteins by five tools (RADAR, TRUST, HHRep, HHRepID, and PTRStalker), with PTRStalker, TRUST, and HHRepID being able to successfully uncover such dimeric structures in some of the tested proteins. In Jorda and Kajava (2009), four methods (T-REKS, XSTREAM, Internal Repeat Finder, and TRED) are compared by the number of sequences they could identify as holding a PTR longer than 14 AA in the SWISSPROT database, with T-REKS giving the highest number (almost doubling the closest competitor). In Marsella et al. (2009), three methods (REPETITA, TRUST, and RADAR) are compared to assess their ability in guessing the correct periodicity in solenoid repeats, with REPETITA having an edge over the other two methods.

## Protein-TR Detection Algorithms Based on Structure

Functional features are more readily linked to the structural features of a protein rather than to their primary sequence, thus available structural data should also be used to detect protein 3d symmetries and repetitive 3d motifs (Goodsell and Olson, 2000). However, only for a fraction of the known protein sequences, the corresponding 3D conformation could be determined, therefore the range of applicability of structure-based methods is limited w.r.t. the range of the sequence-based methods.

In this case, the algorithmic challenge lies in the multidimensional nature of the data, and on the fact that the space of rigid transformations (rotations, translations) as well as the inherent flexibility of proteins must be taken into account when attempting to match 3d substructures in order to detect the PTR periodicity. Table 2 reports the list of structure-based algorithms.

**Table 2:** **Synthetic table of resources for PTR studies: structure-based algorithms**.

Name	Type	Year	Reference	Notes
DAVROS	Alg	2004	Murray et al. (2004)	http://www.ebi.ac.uk/~murray/davros/
OPAAS	Alg	2004	Shih and Hwang (2004)	http://www.ibms.sinica.edu.tw/
Swelfe	Alg	2008	Abraham et al. (2008)	http://wwwabi.snv.jussieu.fr/public/Swelfe/
RQA	Alg	2009	Chen et al. (2009)	
Gplus	Alg	2009	Guerler et al. (2009)	http://agknapp.chemie.fu-berlin.de/gplus/
SymD	Alg	2010	Kim et al. (2010)	http://symd.nci.nih.gov/
ProSTRIP	Alg	2010	Sabarinathan et al. (2010)	http://cluster.physics.iisc.ernet.in/prostrip/
RAPHAEL	Alg	2012	Walsh et al. (2012b)	http://protein.bio.unipd.it/raphael/
Frustratometer	Alg	2013	Parra et al. (2013)	http://www.proteinphysiologylab.tk/
ConSole	Alg	2014	Hrabe and Godzik (2014)	http://console.sanfordburnham.org/
PRIGSA	Alg	2014	Chakrabarty and Parekh (2014)	http://bioinf.iiit.ac.in/PRIGSA/

In Murray et al. (2002), both the sequence and the structure signals are integrated within a continuous wavelet transform approach to detect repeating motifs. In particular, the sequence is represented by values of the Kyte–Doolittle hydrophobicity scale, while structure is characterized via the relative accessible surface area. This approach has been shown to be successful on most of the well known types of repetitive motifs.

DAVROS (Murray et al., 2004) is a PTR prediction system that builds upon a structural alignment program (SAP) that evaluates internal structural symmetries via a protein self-similarity matrix and employs a Fourier Transform approach to identify strong signals over the noisy background.

Swelfe (Abraham et al., 2008) finds internal repeats by combining three abstraction levels. Swelfe quickly identifies statistically significant internal repeats in DNA sequence, in the amino acid sequence and in the 3D structures using dynamic programing. The associated web server also shows the relationships between repeating feature at each level and facilitates visualization of the results.

SymD (Kim et al., 2010) is an algorithm that aims at detecting internal spatial symmetries of proteins. It uses the alignment method in Kim et al. (2009) on pairs of structure formed by the target protein and its shifted versions built by all circular permutations of its residues. Although not all PTR give rise to symmetric 3D structures, many do, therefore this approach often indicates the presence of a PTR. Other methods based on this symmetry detection approach are RQA (Chen et al., 2009), OPAAS (Shih and Hwang, 2004), and Gplus (Guerler et al., 2009).

ProSTRIP (Sabarinathan et al., 2010) uses dynamic programing to find similar structural repeats in a protein structure encoded by the protein backbone dihedral angles.

RAPHAEL (Walsh et al., 2012b) is a more recent method for the detection of solenoids in protein structures. It aims at mimicking the periodicity and distance patterns detection criteria a human curator is likely to exploit when assessing the presence of a solenoid visually. In particular, the candidate protein is subject to a random rotation and translation, and subsequently for each of the three C-alpha coordinates a projection is performed. This operation produces a profile curve, in which the distance between consecutive local maxima is a candidate periodicity value. By averaging over multiple random rotations and translations, a robust period estimation is attained. Additional simple rules allow to further detect non-periodic residues interspersed in the solenoid periodic structure.

Parra et al. (2013) use the structural alignment tool TopMatch (Sippl, 2008) to search exhaustively the space of possible sub-structures that tile a large fraction of a given structure, and thus can represent a *bona fide* structural repetitive element of the input protein.

PRIGSA (Chakrabarty and Parekh, 2014) represents distance information among residues in an adjacency matrix, and it is based on the observation that similar sub-structures can be recognized as unique profiles of the principal eigenspectra of this matrix.

ConSole (Hrabe and Godzik, 2014) aims at detecting solenoid domains having as input structural information, by searching repetitive patterns in a *contact matrix*, which, for every pair of residues *i,j* in a protein, encodes a value 1 if the two residues have at least a pair of heavy atoms at Euclidean distance below a threshold *t* (set at *t* = 4.5 Å). *Ad hoc* rules are further applied in order to handle insertions in the solenoid repetitive patterns.

As in the case of sequence-based methods, very few comparative studies among the proposed structure-based tools have been done. In Kim et al. (2010), six methods (DAVROS, OPAAS, Swelfe, RQA, Gplus, and SymD) are compared in their ability to identify characteristic symmetries in fold families from CATH, SCOP, and ASTRAL databases, with SymD having an overall better performance. In Sabarinathan et al. (2010), two methods (ProSTRIP and Swelfe) are compared over well known families of repeat proteins, for the task of detecting periodicity and exact repeat positions. On well known PTR proteins, both methods detect approximatively the correct period, however, ProSTRIP detects more repeating units. On the harder class of multidomain proteins ProSTRIP is also better at guessing the correct periodicity. In Walsh et al. (2012b) five methods (both sequence and structure based) are compared (namely Swelfe, RAPHAEL, REPETITA, TRUST, and RADAR) in their ability to guess the PTR periodicity, with RAPHAEL giving better predictions, when we allow for a slackness of 5 AA in the predicted value. For exact predictions, RAPHAEL, REPETITA, and TRUST are about equivalent.

## Databases for Protein-TR

Information about PTR can be retrieved as annotations in general purpose integrated protein databases. However, such annotations often cover only the well studied PTR, therefore in recent years a number of special purpose repositories have been assembled with the objective of making large scale PTR analysis easier. We list here in Table 3 only DBs that are available on-line at the present time, as many older published articles refer to DBs no longer available.

**Table 3:** **Synthetic table of resources for PTR studies: databases**.

Name	Type	Year	Reference	Notes
RepSeq	DB	2007	Depledge et al. (2007)	http://www.repseq.org/
PRDB	DB	2012	Jorda et al. (2012)	http://bioinfo.montp.cnrs.fr/
ProRepeat	DB	2012	Luo et al. (2012)	http://prorepeat. bioinformatics.nl/
PTRStalkerDB	DB	2012	Pellegrini et al. (2012)	http://bioalgo.iit.cnr.it/
RepeatsDB	DB	2013	Di Domenico et al. (2013)	http://repeatsdb.bio.unipd.it/

RepSeq (Depledge et al., 2007) is a specialized DB for PTR in lower eukaryotic pathogens.

PRDB is a PTR database that supports queries on protein tandem repeats found in sequence data bases. Currently, it holds about 1.25M PTR extracted from the Swissprot, PDB, and NR databases in early 2010 using the T-REKS detection tool (Jorda et al., 2012). This database has been instrumental for uncovering original biological correlations in Jorda et al. (2010).

PTRStalkerDB lists the PTR found with the PTRStalker method on the SwissProt database release 57.15 of March 2, 2010 that contains 515,203 sequence entries.

ProRepeat (Luo et al., 2012) is a curated and integrated data base and analysis platform for research on the biological features of amino acid tandem repeats. ProRepeat collects PTR of protein sequences listed in the UniProt knowledge base from different species; moreover, it includes 85 completely sequenced eukaryotic proteomes from the RefSeq collection. The latest datasets used in ProRepeat are UniProtKB Release May 2011 and RefSeq Release 40.

RepeatsDB (Di Domenico et al., 2013) is a database of annotated tandem repeat protein *structures* that uses both a state of the art detection method (RAPHAEL) and manual curation to survey the protein structures listed in PDB. The latest version 2.0.0 (beta) released in 2015 holds 10,039 PTR structures (including manually classified and predicted PTR). Automated updates every 3 months are planned.

Although progress in the area of databases for PTR has come about in the past few years, there is also much scope for improvement, in particular, as the amount of proteomic data increases rapidly, it is important to maintain the PTR databases aligned with the latest releases of the reference protein sequence and structure. Also, given the variety of algorithms and approaches to PTR prediction, DB that uses one single algorithm as source of data could suffer for the specific algorithm’s biases, and more robust prediction could be obtained instead by using multiple detecting algorithms.

## Classification of Protein-TR

Kajava (2012) reports an extensive survey of bioinformatic tools to support various analysis of TR in proteins, including tools for identification of TR in proteins, databases reporting PTR (either exclusively, or as an annotation in a larger protein DB), classification of repetitive 3D structures, and tools for structural prediction targeting proteins with PTR (as opposed to globular ones).

Early surveys by Marcotte et al. (1998), Andrade et al. (2001), and Kajava (2001) are very much concerned with the task of identifying specific classes of proteins highly characterized by their PTR content with the aim of finding corresponding structural and functional regularities. Andrade et al. (2001) propose a taxonomy of six main classes (β-propellers, β-trefoils, TPR-like, Ankyrin-like, Armadillo/HEAT-like and Leucine-Rich). Instead Kajava (2001) uses a classification based on the repeating unit length (1–2 residues = class I crystalline aggregates, 3–4 residues = class II fibrous proteins, 5–40 residues = class III solenoid-like proteins, and class IV beads-on-string proteins with repeats longer than 30 residues folded into globular domains). Later in Kajava (2012), a refinement of this classification by splitting class III into two sub-classes of *solenoid* and *non-solenoid* structures has been proposed. The database RepeatsDB (Di Domenico et al., 2013) uses the classification proposed by Kajava (2012).

## Mechanisms of Protein-TR Expansion during Evolution

Björklund et al. (2006) and Moore et al. (2008) analyze the internal sequence similarity in proteins of several species and note that the domain repeats are often expanded through simultaneous duplications of several domains in one event, while the duplication of one domain at a time is a less common event. Moreover, many of the repeats appear to have been duplicated in the middle of the repeat region. This behavior is in contrast to the evolution of other proteins that mainly happens through additions of single domains at either terminus of the protein. No common mechanism for the expansion of all repeats could be detected in this study, for example, duplication patterns show no dependence on the size of the domains. Repeat expansion in some families can possibly be explained by shuffling of exons but exon shuffling does not appear to be a general formation mechanism.

Some domain families show distinct specific duplication patterns, for example, nebulin domains have mainly been expanded with groups of seven domains at a time, while duplications of other domain families involve varying numbers of domains for each event. A more detailed analysis of nebulin domains evolution is in Björklund et al. (2010).

By mapping the Protein TR back onto their coding DNA sequences, Street et al. (2006) study the conservation of intron/exon patterns across several species and show evidence that subdivide the repeat protein genes into two classes. The first class has random-length exons that are likely produced by accumulating introns though random insertion within the array of repetitive units. The second class is composed exclusively of exons corresponding to the multiple of the repeating unit, and thus is likely to be formed by local duplications of intron/exon modules.

## Protein-TR Evolutionary Conservation

In Schaper et al. (2014), it is described a proteome-wide analysis of the evolution of TR in human proteins, using a database of 61 eukaryotes. The main finding is that the vast majority of human PTR are ancient, with TR unit number and order preserved intact since remote speciation events. Moreover, no human PTR shows evidence of a recent duplication or deletion event. Thus, presumably, most PTRs fold into stable and conserved structures, indispensable for their function. Similar findings for plants are shown in Schaper and Anisimova (2015). The analysis of PTR in *Drosophila melanogaster* reported in Ponting et al. (2001) led to the identification of novel PTR in the products of disease-related human genes homologous to those in *Drosophila melanogaster*.

## Protein-TR in Protein Design

Different structures which arise from tandem arrays of a repeated structural motif have generated significant interest with respect to protein engineering and synthetic protein design (Forrer et al., 2003, 2004; Main et al., 2003, 2005; Javadi and Itzhaki, 2013). Several results are reported in these articles about re-engineering of PTR binding specificities, with attention to protein folding kinetics and protein stability.

Sawyer et al. (2013) present a “module-based” design approach in which modules composed of tandem repeats are aligned to identify repeat-specific features that will be important to include in future repeat protein design templates.

Parmeggiani et al. (2015) describe a general database-driven approach for reliable generation of synthetic stable modular repeat proteins. Concomitant to the distillation of general design principles for PTR engineering, research activities have been also concentrated toward specific classes of Protein-TR which have shown a more promising potential for applications (Stumpp et al., 2015). A notable example is that of *Designed Ankyrin Repeat Proteins (DARPins)* (Binz2003) that have been extensively studied [see a recent survey by Plückthun (2015) and references therein], since they provide a biochemically stable scaffold for designing protein variants able to recognize targets with affinity and specificity that are equal or possibly superior to that of antibodies. Similar promising studies focus also on *armadillo repeat proteins* (Reichen et al., 2014) and *leucine-rich-repeat proteins* (Park et al., 2015).

## Order, Disorder, and Protein-TR

While our view of protein functions is often linked to the presence of a well defined 3-dimensional protein conformations, it has been recognized (Tompa, 2002) that many important protein functions are also linked to proteins (or regions within a protein) that lack a folded structure, but display a highly flexible random-coil-like conformation under physiological conditions [named intrinsically unstructured proteins (IUP) or intrinsically unstructured regions (IUR)].

The concept of order and disorder in protein segments (Dunker et al., 2001; Tompa, 2002) has been often investigated in correlation with the presence or absence of PTR at the sequence level. For example in Tompa (2002), 21 IUP are examined, and further 21 cases are cited in Dunker et al. (2001). It is noticed that IUR often correspond to regions of low compositional complexity (low sequence entropy) and sometimes to repetitive sub-sequences in fibrillar proteins. Tompa and Fersht (2009) discuss in detail the cases of PTR in PEVK regions of human Titin, in prion proteins and in the CTD domain of RNA polymerase. These findings on specific instances are, however, hard to generalize.

A general property observed by Jorda et al. (2010) is that higher level of repeat perfection correlates positively with the disordered state of protein sub-chains.

The emergence of IUP/IUR prediction tools, such as IUpred (Dosztányi et al., 2005), ESpritz (Walsh et al., 2012a), and DISOPRED (Jones and Cozzetto, 2015), to name a few, and comprehensive databases of IUP/IUR, such as DisProt (Sickmeier et al., 2007) and MobiDB 2.0 (Potenza et al., 2015), can be quite useful for finding generalizable connections between PTR and ordered/disordered states of protein regions.

## Correlation of Protein-TR with Other Protein Properties

In Turutina et al. (2006), the sequences of the *Swiss-Prot* protein families are analyzed in order to detect family-specific latent periodicity fingerprints induced by PTR, using the method in Korotkov et al. (2003), and 94 such protein families are reported as well-characterized by such fingerprints.

A complete analysis of PDB sequences using RADAR is reported in Rajathei and Selvaraj (2013), where a good correlation among PTR, structural similarity, and functionally involved residues is highlighted.

In Mularoni et al. (2007) and Mularoni et al. (2010), the function and evolution of a particular class of PTR formed by repetitions of a *single AA* are investigated (homo-TR). These two studies concentrated on human and mouse homo-TR of length four. The protein stabilizing properties of homo-TR are also reported in Katti et al. (2000). A more general statistical analysis of homo-PTR in human proteins is in Jorda and Kajava (2010).

## Conclusion

The present survey on Protein-TR touches several aspects of this research fields, including detection algorithms (Sections “Protein-TR Detection Algorithms Based on Sequence” and “Protein-TR Detection Algorithms Based on Structure”), databases (Section “Databases for Protein-TR”), classification (Section “Classification of Protein-TR”), the relationship between PTR and biologically relevant concepts (Sections “Mechanisms of Protein-TR Expansion During Evolution,” “Protein-TR Evolutionary Conservation,” “Order, Disorder, and Protein-TR,” and “Correlation of Protein-TR with Other Protein Properties”), and it highlights also recent progress in the design of synthetic PTR (Section “Protein-TR in Protein Design”).

Although there has been steady progress in the last 15 years in devising new prediction tools, both sequence and structure based, very little comparative or integrative work has been done. Most of the proteome-wise studies use only one tool to define and detect PTR and draw conclusions on PTR distributions and statistics. Though this approach was completely justified in the pioneering times (late 1990s and early 2000s), it is necessary now to refine these methodologies and make full use of the wealth of algorithms and approaches devised in the last decade. A more robust assessment of the distribution and annotations of PTR over the entire proteome could be attained by applying and merging the outcomes of multiple tools. In this context, the manually curated databases of PTRs can provide the necessary validation benchmarks.

From the point of view of the design of prediction tools, one open challenge is to devise sequence-based tools that are able to come close to the performance of structure-based tools. Thus providing higher quality PTR predictions for a larger pool of sequenced proteins.

## Conflict of Interest Statement

The author declares that the research was conducted in the absence of any commercial or financial relationships that could be construed as a potential conflict of interest.

## Funding

The present work is partially supported by the Flagship project InterOmics (PB. P05), funded by the Italian Ministry for Instruction University and Research (MIUR) and CNR organizations, and by the joint IIT-IFC Laboratory of Integrative Systems Medicine (LISM).
